# Grandparental childcare and physical activity among rural older adults in China: evidence from the China longitudinal aging social survey

**DOI:** 10.3389/fpubh.2026.1870262

**Published:** 2026-07-09

**Authors:** Daiming Li, Xiaoyun Chang, Aidi Liao

**Affiliations:** Department of Political Science and Public Administration, School of Public Administration, Xiangtan University, Xiangtan, Hunan, China

**Keywords:** China, CLASS, grandparental childcare, physical activity, rural older adults

## Abstract

**Background:**

Population aging in rural China is increasing, making the promotion of health among rural older adults an urgent issue. Physical activity is a crucial way to maintain health in later life and has attracted significant attention from researchers both domestically and internationally. Grandparental childcare, as an important form of intergenerational family support, may play a key role in influencing older adults’ participation in physical activity. However, there is limited empirical evidence on the mechanisms that explain these effects. This study explores the association between grandparental childcare and physical activity among rural older adults and further examines potential mechanisms underlying this relationship.

**Methods:**

The study used data from 6,188 respondents in the 2023 China Longitudinal Aging Social Survey. After controlling for sociodemographic factors such as gender, age, education, self-rated health, and income, a binary logistic regression model was applied to examine the association between grandparental childcare and physical activity.

**Results:**

Descriptive statistics show that 60% of rural older adults provide grandparental childcare, and 44.2% participate in physical activity. The regression results indicate that providing grandparental childcare is associated with a 6.66-percentage-point higher probability of engaging in physical activity. Heterogeneity analysis reveals that this association is stronger among women and older adults aged 60–69.

**Conclusion:**

Grandparental childcare is significantly associated with participation in physical activity among rural older adults, with stronger associations observed among women and younger older adults. It is recommended that rural communities integrate physical activity opportunities into daily caregiving contexts, make better use of existing public spaces, and provide health education and caregiver support for older adults who provide grandparental childcare. These initiatives can help support caregiving responsibilities and contribute to healthy aging among rural older adults.

## 1 Introduction

Population aging has become a global trend. It is estimated that the number of people aged 60 and above worldwide will increase from 1 billion in 2020 to 1.4 billion in 2030, and double to 2.1 billion by 2050 (1). In this context, promoting healthy aging has become a key issue in global public health. Regular physical activity is well-known for its health benefits, including improved cardiovascular health, lower risk of chronic diseases, and a significant reduction in all-cause mortality (2–7). For older adults, regular activity is especially important in delaying functional decline, maintaining independence, and enhancing quality of life in later years (8–13). However, physical activity participation among older adults remains suboptimal. The World Health Organization reports that physical inactivity rises sharply after age 60, with older adults engaging in far less exercise than other adult populations (14). In China, only 26.1% of older adults regularly participate in physical activity, and the figure drops to 14.7% for those aged 80 and above. Rural older adults consistently show lower activity levels compared to their urban peers, highlighting a persistent gap in exercise participation between urban and rural areas (15).

Most studies have focused on the factors influencing older adults’ participation in physical activity, examining both individual characteristics and external factors. At the individual level, key factors such as age, gender, education, and income are frequently discussed (16–19). A negative association with age is well established, as physical activity tends to decline progressively with physiological deterioration (20). Gender differences are often attributed to women’s dual roles in family and work, which limit available leisure time (21). Older adults with higher education levels typically have a better understanding of the health benefits of exercise and are more likely to engage in physical activity (22, 23). In contrast, the effect of income on physical activity is more complex and non-linear. Some studies suggest a positive influence (24), while others find no significant relationship (25) or even a potential negative association (26).

Grandparental childcare refers to the daily care and support that grandparents provide for their grandchildren. In rural China, with many young and middle-aged adults migrating to urban areas, a significant number of older adults live in empty-nest, intergenerational, or single-person households. As a result, grandparental childcare has become a common form of intergenerational family support (27–29). Wang and Gonzales (30) found that nearly half of the grandparents engaged in childcare provide an average of 8 h of care per day, totaling more than 40 h per week. Moreover, Weng (31) indicates that participation in the “care-reciprocity” model in rural families is closely related to older adults’ health status. Theoretically, grandparental childcare may be associated with physical activity among older adults through two opposing pathways. On the one hand, according to time constraint theory, caring for grandchildren requires substantial time and energy, which may reduce the time available for physical activity, resulting in a negative effect (32). On the other hand, from the perspective of social support theory, both instrumental and emotional support are important motivators for maintaining positive health behaviors (33–35). Providing childcare may enhance older adults’ self-efficacy, encourage them to maintain a certain level of physical activity, and increase opportunities for outdoor and social activities, thereby indirectly promoting physical activity (36–38).

Although existing studies from individual and social-environment perspectives provide valuable theoretical and empirical insights into physical activity among rural older adults, several areas remain underexplored. First, most research focuses on the influence of individual characteristics on physical activity, with limited attention to the increasingly common phenomenon of grandparental childcare in rural areas. Second, the few studies examining the relationship between grandparental childcare and physical activity are mostly conducted in Western contexts, and evidence based on large-scale data from rural China is scarce. Third, it remains unclear whether the associations between grandparental childcare and physical activity varies across gender, age, or other subgroups. To explore these issues further, this study uses data from the China Longitudinal Aging Social Survey to systematically examine the association between grandparental childcare and physical activity among rural older adults in China and further explore heterogeneity across gender and age groups.

## 2 Methods

### 2.1 Data and sample

This study uses data from the 2023 China Longitudinal Aging Social Survey (CLASS 2023). CLASS is a national and ongoing large-scale social survey designed and conducted by the Institute of Gerontology at Renmin University of China, aiming to comprehensively track the social, economic, health, and family conditions of older adults in China. The survey adopts a multistage stratified probability sampling method and covers 28 provinces, autonomous regions, and municipalities across the country (excluding Hong Kong, Macao, Taiwan, Hainan, Tibet, and Xinjiang). The 2023 wave is the most recent round and provides a good representation of the current living conditions of rural older adults in China. In this study, rural older adults are defined as individuals aged 60 and above with an agricultural household registration (including “agricultural hukou” and “converted from agricultural to unified resident hukou”). The original sample was cleaned through the following steps. First, respondents with rural household registration were selected. Second, only individuals aged 60 years and above were retained. Third, cases with missing values in the dependent variable, independent variable, and key control variables were excluded. After data cleaning, a total of 6,188 valid samples were obtained. The sample covers 28 provinces across China and shows good national representativeness.

### 2.2 Measures and variables

#### 2.2.1 Dependent variable

According to the conceptual definition of physical activity (39), engaging in physical activity less than once per month is considered occasional rather than regular (40, 41). Therefore, such behavior was classified as non-participation. Previous studies have commonly operationalized participation in physical activity as a dichotomous variable when examining physical activity behavior among older adults (42, 43). In this study, participation in physical activity was measured using item G2 of the CLASS questionnaire: “How often do you participate in physical activity?” Respondents who reported participating in physical activity less than once per month were classified as non-participants (0), whereas those who reported participating in physical activity once per month or more were classified as participants (1).

#### 2.2.2 Independent variable

Grandparental childcare is measured using multiple items in the CLASS questionnaire, including whether respondents take care of grandchildren for different children. In this study, a binary variable is constructed to indicate whether respondents provide grandparental childcare. If a respondent answered “yes” to caring for grandchildren for any of their children, they are defined as providing grandparental childcare (coded as 1); otherwise, they are defined as not providing grandparental childcare (coded as 0).

#### 2.2.3 Control variable

To accurately assess the association between grandparental childcare and physical activity, this study includes several sociodemographic and health-related variables as control variables. These include gender (1 = male, 0 = female), marital status (1 = with spouse, 0 = without spouse), age (continuous), education level (continuous), self-rated health (ranging from 1 = “very poor” to 5 = “very good”), and total personal income (continuous).

### 2.3 Statistical analysis method

All statistical analyses were conducted using Stata 18.0. Descriptive statistics were first used to summarize the sample characteristics and the distribution of key variables. Given that the dependent variable, physical activity participation, is binary, a binary logistic regression model was used to assess the association between grandparental childcare and physical activity. A stepwise approach was employed to sequentially add control variables and the main independent variable to test the stability of the coefficients. To aid interpretation, the average marginal effect of the key independent variable was calculated. Subgroup analyses were then conducted to explore potential heterogeneity. Finally, robustness checks were performed using an alternative Probit model and propensity score matching (PSM). Statistical significance was set at *p* < 0.05.

## 3 Results

### 3.1 Descriptive results

Table 1 presents the descriptive statistics of the sample. The average age of respondents was 71.68 years, with most participants being seniors, ranging from 60 to 98 years. The sample included 53.01% men and 46.99% women, showing a relatively balanced gender distribution. The average self-rated health score was 3.49, falling between “fair” and “good,” which indicates a slightly above-average health status. Among respondents, the largest proportion rated their health as “good” (43.74%), while 8.29% reported their health as “poor” or “very poor.”

**Table 1:** Descriptive statistics of rural older adults (*N* = 6,188).

Variable	*n*	%/*M* (SD)
Age (years)		
60–64	592	9.57
65–69	1,701	27.49
70–74	2,277	36.80
75–79	957	15.47
80+	661	10.68
Gender		
Male	3,280	53.01
Female	2,908	46.99
Education (years)	6,188	4.44 (3.15)
Marital status		
Married	4,936	79.77
Unmarried/widowed	1,252	20.23
Personal annual income (log)	6,188	9.22 (1.16)
Self-rated health		
Very poor	72	1.16
Poor	441	7.13
Fair	2,440	39.43
Good	2,706	43.73
Very good	529	8.55
Grandparental childcare		
Yes	3,713	60.00
No	2,475	40.00
Participation in physical activity		
Yes	2,735	44.20
No	3,453	55.80

Continuous variables are reported as mean (standard deviation), categorical variables as frequency (percentage).

The average education level of rural older adults was 4.44 years, reflecting the generally low educational attainment in this group. Among the 6,188 rural older adults, 44.20% engaged in physical activity, suggesting that more than half did not maintain regular activity habits. Additionally, 60.00% of rural older adults provided grandparental childcare, highlighting the prevalence of intergenerational caregiving in rural areas. This trend is closely linked to the structure of rural Chinese families and the significant migration of younger labor to urban areas, indicating that grandparental childcare plays a key role in family support across generations.

### 3.2 Regression results

Table 2 presents the results of the logistic regression analysis that examines the association between grandparental childcare and physical activity among rural older adults. A stepwise regression approach was applied. Model 1 includes only the control variables, while Model 2 additionally incorporates the key independent variable, grandparental childcare. The correlation matrix of all control variables is reported in Supplementary Table S1. The absolute values of all pairwise correlations are below 0.33, indicating that multicollinearity is unlikely to be a serious concern.

**Table 2:** Logistic regression results of grandparental childcare on participation in physical activity.

Variable	Model 1 b (SE)	Model 2 b (SE)	Marginal effect b (SE)
Age	−0.027***	−0.026***	−0.006***
	(0.005)	(0.005)	(0.001)
Gender (Male = 1)	0.079	0.088	0.021
	(0.052)	(0.053)	(0.013)
Education (years)	0.022**	0.023***	0.006***
	(0.009)	(0.009)	(0.002)
Marital status (Married = 1)	0.072	0.078	0.019
	(0.073)	(0.073)	(0.018)
Self-rated health	0.041	0.041	0.010
	(0.033)	(0.033)	(0.008)
Personal annual income (log)	−0.067***	−0.075***	−0.018***
	(0.023)	(0.023)	(0.006)
Grandparental childcare (Yes = 1)	—	0.274***	0.067***
	—	(0.053)	(0.013)
Constant	2.034***	1.845***	—
	(0.456)	(0.459)	—
Observations	6,188	6,188	6,188
Pseudo *R*^2^	0.010	0.013	—

*p < 0.1, **p < 0.05, ***p < 0.01. Standard errors are reported below coefficients in parentheses.

Results from Model 1 show that age is significantly negatively associated with physical activity (*β* = −0.027, *p* < 0.001), indicating that the likelihood of participating in physical activity decreases with increasing age. Years of education are significantly positively associated with (*β* = 0.022, *p* < 0.05), suggesting that rural older adults with higher education levels are more likely to engage in physical activity. In contrast, annual personal income shows a significant negative association with physical activity (*β* = −0.067, *p* < 0.001). In addition, the effects of gender, marital status, and self-rated health are not statistically significant (*p* > 0.1 for each).

Model 2 further includes the key independent variable, grandparental childcare. The results show that grandparental childcare has a significantly positive coefficient (*β* = 0.274, *p* < 0.001), indicating that, after controlling for other factors, grandparental childcare is significantly associated with a higher likelihood of participating in physical activity than those who do not. The marginal effect indicates that providing grandparental childcare is associated with a 6.66-percentage-point higher probability of participating in physical activity. The direction and significance of the control variables remain largely stable.

### 3.3 Heterogeneity test

To examine group differences in the association between grandparental childcare and physical activity, subgroup regression analyses were conducted, and the results are presented in Table 3. By gender, the positive association between grandparental childcare and physical activity is stronger among women. The marginal effect is 7.73% (*p* < 0.001) for women, compared with 5.60% (*p* < 0.01) for men. By age, the association is slightly stronger for younger older adults (60–69 years) than for those aged 70 and above. The marginal effect is 7.48% (*p* < 0.001) for the younger group and 6.62% (*p* < 0.01) for the older group.

**Table 3:** Heterogeneity analysis: marginal effects of grandparental childcare on participation in physical activity.

Group	AME (%)	Standard error	*p*-value	Observations
Gender				
Female	7.73	1.90	<0.001	2,908
Male	5.60	1.80	0.002	3,280
Age				
60–69	7.48	2.00	<0.001	2,789
70+	6.62	1.80	0.001	3,399

Marginal effects estimated from subgroup logistic regression models.

## 4 Robustness test

To ensure the robustness of the findings, robustness checks were conducted from two aspects: model specification and sample selection bias.

### 4.1 Alternative model specification

The baseline model was re-estimated by replacing the Logit model with a Probit model. The direction and significance of the coefficient for grandparental childcare remain largely unchanged, indicating that the baseline results are not sensitive to the model specification.

### 4.2 Propensity score matching

Since providing grandparental childcare is not randomly assigned, healthier older adults may be more likely to take on caregiving responsibilities, which may lead to self-selection bias in the baseline regression. To address this issue, propensity score matching (PSM) was applied to construct a counterfactual framework. Gender, age, years of education, marital status, self-rated health, and the logarithm of annual income were included as covariates to estimate each individual’s propensity to provide grandparental childcare. A 1:1 nearest neighbor matching method with a caliper of 0.05 was used. The balance test results (Table 4) show that, after matching, the absolute values of the standardized bias for all covariates are below 10%, indicating good matching quality and no systematic differences in observable characteristics between the treatment and control groups. Although the standardized difference for education increased slightly after matching (from 0.031 to 0.083), it remained below the commonly accepted threshold of 0.10, indicating that balance was still achieved. Similar patterns have been reported in nearest-neighbor matching procedures when matching quality improves overall but individual covariates show minor fluctuations. The estimated ATT after matching is reported in Table 5. Providing grandparental childcare is associated with a 6.5-percentage-point higher probability in physical activity among rural older adults. This estimate is very close to the marginal effect from the baseline regression (6.66%), suggesting that the positive association between grandparental childcare and physical activity remains robust even after accounting for self-selection bias.

**Table 4:** Covariate balance before and after propensity score matching.

Variable	Standardized difference (raw)	Standardized difference (matched)	Bias reduction (%)
Gender (Male = 1)	−0.058	0.004	92.6
Age	−0.185	0.032	82.7
Education (years)	0.031	−0.083	—
Marital status (Married = 1)	0.205	−0.023	88.8
Self-rated health	0.058	0.011	81.1
Personal annual income (log)	0.188	0.021	88.8

Standardized differences are reported in absolute values. Following common practice in propensity score matching studies, absolute standardized differences below 0.10 are considered indicative of satisfactory covariate balance.

Propensity score matching substantially improved the balance of most covariates between the treatment and control groups. Although the standardized difference for education increased slightly after matching, its absolute value remained below the conventional threshold of 0.10. Moreover, all covariates satisfied the commonly used balance criterion after matching, suggesting that the matching procedure achieved an acceptable level of covariate balance overall.

**Table 5:** Average treatment effect (ATT) of grandparental childcare on physical activity participation after propensity score matching.

Matching method	ATT	Standard error	*z*-value	*p*-value
1:1 nearest neighbor (caliper = 0.05)	6.50	1.80	3.68	<0.001

ATT represents the average effect of providing grandparental childcare on the probability of participating in physical activity among rural older adults. Standard errors estimated using 1:1 nearest neighbor matching with caliper 0.05.

### 4.3 Alternative classification of physical activity

To further examine the robustness of the findings, the dependent variable was redefined using a more stringent threshold. Specifically, respondents who reported engaging in physical activity at least once per week were classified as participants (1), whereas those engaging in physical activity less than once per week were classified as non-participants (0). The logistic regression model was then re-estimated using this alternative classification.

The results indicate that grandparental childcare remains significantly associated with physical activity participation among rural older adults. The average marginal effect of grandparental childcare was 0.0496 (*p* < 0.001), indicating that older adults providing grandparental childcare had a 4.96-percentage-point higher probability of participating in physical activity under the stricter weekly threshold. Although the estimated marginal effect is slightly smaller than that reported in the main analysis (6.66%), the direction and statistical significance remain unchanged. These findings suggest that the main results are robust to alternative definitions of physical activity participation.

## 5 Discussion

Age is significantly negatively associated with physical activity participation, a pattern explained by multiple interacting factors. Physiologically, long-term agricultural work often leads to joint strain and chronic pain, which directly limit physical capacity (44, 45). Psychologically, the absence of positive exercise experiences may create a cycle of low self-efficacy among older rural adults, where a lack of confidence further discourages them from being active. Physical labor is often regarded as the only legitimate form of physical exertion, while voluntary physical activity may be seen as unnecessary or inappropriate (46, 47). Socially, shrinking networks in later life reduce peer support and access to role models. Additionally, cultural norms that encourage older adults to rest and avoid exertion further diminish external motivation to engage in physical activity (48–50).

The positive association between education and physical activity is consistent with previous studies. Older adults with higher levels of education have a clearer understanding of the health benefits of physical activity and show stronger intrinsic motivation to participate. At the same time, their more favorable socioeconomic conditions, including more stable income sources and better living environments, provide more convenient conditions and greater social support for engaging in physical activity (51).

Marital status does not significantly affect physical activity participation. One possible explanation is that spousal support does not always positively influence exercise behavior, and a poor-quality marital relationship may even have a negative impact (52). In rural China, older adults without a spouse often live with their children or grandchildren. These close intergenerational relationships can replace the companionship and emotional support typically provided by a spouse, thus buffering the potential negative effect of spousal absence on physical activity.

Self-rated health does not show a significant association with physical activity. One possible explanation is that activity decisions among rural older adults are largely influenced by external factors, such as the social role of caring for grandchildren and the physical demands of agricultural work. As a result, subjective health perceptions have a relatively limited impact on their participation in physical activity.

Personal annual income shows a significant negative association with participation in physical activity. Although this finding may seem counterintuitive, it aligns with the “income–leisure substitution” hypothesis. In rural contexts, higher income may be accompanied by longer working hours and greater production demands, which reduce the time and energy available for additional exercise (53). Because the CLASS dataset does not contain detailed measures of daily labor hours or agricultural work intensity, the mechanism linking income to lower physical activity cannot be directly verified. These results suggest that increases in income alone may not be sufficient to raise physical activity levels among rural older adults, and that policy interventions should also account for constraints related to labor intensity and available leisure time.

This study further finds that grandparental childcare is significantly associated with physical activity among rural older adults. Several hypothesized pathways may help explain the observed association between grandparental childcare and physical activity among rural older adults. It should be noted that these pathways are theoretical interpretations derived from previous literature and were not empirically tested in the present study. First, a potential psychological motivation pathway, where the responsibility of caring for grandchildren is associated with a stronger sense of self-efficacy and family value. This positive psychological state may correlate with an internal drive to maintain physical function, which may be related to greater participation in physical activity (54, 55). Second, a potential social expansion pathway, where providing childcare often requires older adults to accompany grandchildren to community squares, parks, and children’s activity areas. This exposure to public spaces naturally increases their contact with other exercisers and fitness facilities, which may activate peer effects within social networks (56, 57). Third, a potential emotional support pathway, where frequent intergenerational interaction and close companionship provide stable positive emotional feedback for older adults. This emotional support helps reduce feelings of loneliness and sustains their willingness to engage in outdoor activities and social participation, which may be associated with higher levels of physical activity (58). These mechanisms may help explain the observed association in physical activity among rural older adults. It should be noted that the present study did not directly examine these pathways empirically. Therefore, the proposed explanations should be interpreted as theoretical hypotheses rather than verified mechanisms. However, previous studies suggest that the health effects of grandparental childcare may show a “dose–nonlinear” pattern (59). Future research using continuous measures of childcare intensity may help to clarify the boundary conditions of the positive associations identified in this study.

The heterogeneity analysis reveals group differences in the associations between grandparental childcare and physical activity. First, gender differences are observed. The marginal effect for women (7.73%) is notably higher than that for men (5.60%), which is consistent with the traditional gender division of roles in rural society. In rural China, women typically take on more childcare responsibilities within the family (60), and thus grandparental childcare leads to more substantial changes in their daily routines. This finding is in line with the study by Notara, which emphasizes that social connections play an important role in promoting participation in physical activity (61), and women are generally more active in building and maintaining such connections. Second, age differences are also evident. The positive association between grandparental childcare and physical activity is slightly stronger among younger older adults aged 60–69 than among those aged 70 and above, reflecting differences in physical capacity. Younger older adults are in better physical condition and are more able to combine caregiving with physical activity. In contrast, caregiving among older age groups is more likely to involve indoor supervision, which provides fewer opportunities to promote physical activity.

Several limitations should be acknowledged when interpreting the findings of this study. First, the cross-sectional design and the use of propensity score matching prevent causal inference. While matching reduces self-selection bias from observable variables, unobserved characteristics, such as personality and health awareness, may still confound the results. Second, grandparental childcare and physical activity were measured as binary variables, which do not capture differences in intensity, duration, or dynamic changes over time. Third, agricultural labor intensity and work status were not available in the dataset, which may confound the observed association between grandparental childcare and physical activity in rural older adults. Fourth, physical activity was assessed using a single self-reported item, whose psychometric properties are unknown, and which does not capture activity type, intensity, or duration. Therefore, the findings should be interpreted as associations rather than causal effects. Future studies could employ continuous or validated measures of physical activity and include detailed information on work and caregiving intensity to provide a more nuanced understanding of physical activity behavior among rural older adults.

## 6 Conclusion

This study draws the following main conclusions. First, grandparental childcare is significantly associated with participation in physical activity among rural older adults. After controlling for individual characteristics, socioeconomic status, and health conditions, older adults who provide grandparental childcare show a 6.66-percentage-point higher probability of participating in physical activity than those who do not, providing further evidence of the observed association without implying causality. Second, the association between grandparental childcare and physical activity varies across subgroups: it is more pronounced among women and younger older adults aged 60–69, highlighting differences related to gender and age without inferring causal mechanisms.

Based on these findings, rural health promotion programs may consider integrating physical activity opportunities into daily caregiving contexts for older adults who provide grandparental childcare. Rather than relying primarily on new infrastructure, communities can make better use of existing public spaces and organize low-cost intergenerational activities, health education, and caregiver support programs. In addition, strengthening social support networks and family support for older caregivers may help reduce caregiving burden while encouraging safe and age-appropriate physical activity among rural older adults. Such strategies may support grandparental childcare responsibilities while contributing to healthy aging.

## Data Availability

Publicly available datasets were analyzed in this study. This data can be found at: https://class.ruc.edu.cn.
