# Supplementary material for: Grandparental childcare and physical activity among rural older adults in China: evidence from the China longitudinal aging social survey
Source: Front Public Health. 2026 Jul 9;14:1870262. doi: 10.3389/fpubh.2026.1870262 (PMC13391402; doi:10.3389/fpubh.2026.1870262)
Supplement: Supplementary file 1 [file Table_1.docx]

**Supplementary Table S1. Pearson correlation matrix of control variables**

| **Variable** | **Gender** | **Age** | **Marital status** | **Education** | **Self-rated health** | **Personal annual income** |
| --- | --- | --- | --- | --- | --- | --- |
| **Gender** | **1.000** |  |  |  |  |  |
| **Age** | **-0.020** | **1.000** |  |  |  |  |
| **Marital status** | **0.128** | **-0.258** | **1.000** |  |  |  |
| **Education** | **0.161** | **-0.284** | **0.177** | **1.000** |  |  |
| **Self-rated health** | **0.063** | **-0.157** | **0.095** | **0.081** | **1.000** |  |
| **Personal annual income** | **0.069** | **-0.168** | **0.122** | **0.156** | **0.100** | **1.000** |
